# Follow-Up of Low-Acuity Patients After Redirection From a Swiss Emergency Department Using an Electronic Triage Application: Protocol for a Single-Center Prospective Cohort Study

**DOI:** 10.2196/82195

**Published:** 2026-03-27

**Authors:** Flora Gobet, Anne-Laure Feral-Pierssens, Thomas Castelain, Ludovic Galofaro, Hugo Najberg, Vincent Ribordy, Youcef Guechi

**Affiliations:** 1 Faculty of Medicine, University of Fribourg, Chemin du Musée 8, Fribourg, 1700, Switzerland, +41 26 306 31 42; 2 Department of Emergency Medicine, Fribourg Hospital, Chemin des Pensionnats 2/6, Villars-sur-Glâne, 1752, Switzerland, +41 26 306 31 42; 3 Department of Emergency Medicine, Avicenne Hospital, Public Assistance Hospitals Paris, Bobigny, France; 4 Laboratory for Education and Health Promotion, University Paris-Sorbonne, Villetaneuse, France; 5 Department of Anesthesiology and Intensive Care, Hospital Oberwallis, Visp, Switzerland; 6 Directorate of IT Services, University of Fribourg, Fribourg, Switzerland

**Keywords:** emergency service, low-acuity emergency department visit, low-acuity ED visit, nonurgent emergency department visit, nonurgent ED visit, patient acuity, triage, referral and consultation, redirection, LGI Emergency Redirection, health literacy, crowding

## Abstract

**Background:**

Emergency department (ED) overcrowding threatens health care systems worldwide. This poses risks to patient safety, lowers quality of care, and reduces patient satisfaction. Patient input, defined as the caseload of patients presenting to the ED, is one of the factors contributing to overcrowding. Redirecting patients with nonurgent complaints to external health care services could help alleviate ED workload.

**Objective:**

The primary objective of this study is to investigate the safety and efficiency of redirection of low-acuity patients using an electronic clinical decision support system. As a secondary and exploratory aim, we will assess the impact of this intervention on subsequent health care use, patient satisfaction, and health literacy over a 6-month follow-up period.

**Methods:**

A single-center observational study with 2 consecutive cohorts of low-acuity patients presenting to the ED will be conducted. The first cohort will be triaged and offered redirection according to current practice. In the second cohort, after triage, low-acuity patients will be evaluated by an electronic clinical support system to determine eligibility for redirection. If eligible for redirection, an appointment at a nearby clinic will be arranged through the system. The primary end point is any unexpected returns to health care services within 48 hours of triage. Secondary end points are patient satisfaction as well as the number of unexpected consultations and the evolution of health literacy during a 6-month follow-up period.

**Results:**

This study is funded by an internal grant from Hôpital Fribourgeois. Recruitment began on June 20, 2025, with a total of 35 patients enrolled as of August 2025. Data analysis will begin after the recruitment and 6-month follow-up of 300 to 420 patients are completed, which is expected to occur in November 2026. We hope to publish results in December 2026.

**Conclusions:**

We expect the redirection process of low-acuity patients to other health care facilities using an electronic clinical support system to be safe and efficient. If results are positive, application of this reproducible strategy could reduce the number of patients treated in EDs and provide alternative health care pathways for low-acuity patients.

## Introduction

Emergency department (ED) overcrowding is a global issue with significant consequences for both quality of care and the overall performance of health care systems. EDs operating overcapacity have been associated with poorer patient outcomes, increased mortality, decreased patient satisfaction, elevated staff stress, and lower adherence to clinical guidelines [1,2]. A 3-level model (input, throughput, and output) has been proposed to describe the causes and potential solutions to ED overcrowding [3]. While most interventions aim to improve patient flow by focusing on efficiency during a patient’s ED stay, interventions focusing on patients before ED admission remain largely understudied [4].

In parallel, high numbers of low-acuity patients increase ED workload and may divert resources from critically ill or high-complexity cases. The use of EDs by low-acuity patients can be partially linked to limited access to timely primary care consultations [2,5,6]. Furthermore, low health literacy also contributes to inappropriate use of EDs and is linked to poorer ED outcomes [7-9]. Consequently, low-acuity patients represent a potentially modifiable aspect of ED input, and redirecting those who do not require hospital-based care to external medical services [10] has emerged as a proposed strategy to reduce ED crowding.

Although redirection alone is not sufficient to resolve the underlying structural causes of ED overcrowding, which include a lack of capacity in outpatient services and access block [1], it may contribute to improving patient experience and local workflow. Perceived overcrowding and long wait times are directly associated with a significant decline in real-time patient satisfaction [11]. Redirection alleviates this problem by offering patients with minor conditions a faster alternative, preventing unnecessary waiting time in highly specialized medical centers, while allowing resources to be focused on patients who need them most [12,13]. This restructuring also benefits staff, as excessive congestion is a major cause of emotional exhaustion and job dissatisfaction. Therefore, lightening the load of nonurgent cases can improve working conditions for clinicians.

Redirection criteria must reliably and reproducibly identify patients whose needs can be met by external health care providers. Traditional ED triage systems are limited as they classify patients by vital risk and urgency to determine priority in admission and do not determine eligibility for redirection [14,15]. Previous research at a university hospital in Montreal demonstrated that an electronic clinical support system (LGI Emergency Redirection) could safely redirect patients, with an unexpected return rate below 5% and improvements in ED performance indicators [16,17].

Nonurgent consultations in the ED have been on the rise in the last decade in Switzerland, with more patients being self-referred, and the most frequently reported reason for self-referral is a lack of awareness of alternative care options[6]. There is growing interest in examining the feasibility of selectively redirecting low-acuity patients to alternative health care services [18]. To this end, we aim to assess the safety of implementing an electronic clinical support tool to identify patients eligible for redirection from a Swiss ED to external health care providers. To our knowledge, this is the first time such a redirection strategy has been studied in Switzerland. We aim to show that this strategy will be safe and will prove to be an effective decision support addition to current triage practice. Furthermore, we aim to evaluate the impact of this structured redirection on patient satisfaction, subsequent unplanned consultations, and health literacy, predicting that the formal appointment scheduling provided by the system will lead to improved longitudinal quality of care.

## Methods

### Study Design and Setting

This study will be a single-center, prospective observational cohort study focused on selective redirection of low-acuity patients, as determined by the electronic clinical support system (LGI Emergency Redirection, LGI Healthcare Solutions). Recruitment will take place between June 2025 and May 2026. The study will consist of 2 consecutive phases, detailed below. Each phase will last approximately 4 to 6 months, with a planned follow-up period of 6 months after the initial visit. The total expected study duration is approximately 15 months.

Participants will be recruited during triage at the ED of the Cantonal Hospital of Fribourg in Switzerland. This is a regional multidisciplinary adult ED managing around 41,000 annual ED visits.

Triage is based on the revised Swiss Emergency Triage Scale (SETS), which categorizes patients into 4 urgency levels based on presenting complaint, medical history, and vital signs [19]. Critical conditions requiring immediate medical attention are classified as SETS level 1, while SETS level 4 represents nonurgent conditions. Some presenting complaints can be classified at multiple levels depending on vital signs and medical history (eg, cardiac arrest=1, chest pain=1 or 2, abdominal pain=2 or 3, and dysuria=3 or 4). Since 2022, our ED has redirected level 4 patients by advising them to consult alternative health care providers (eg, walk-in clinics and general practitioners [GPs]). The safety of this approach has been established in a previous study [20].

### Electronic Clinical Decision Support System: Operational Steps and Case Scenario

#### Overview

**Figure 1.**
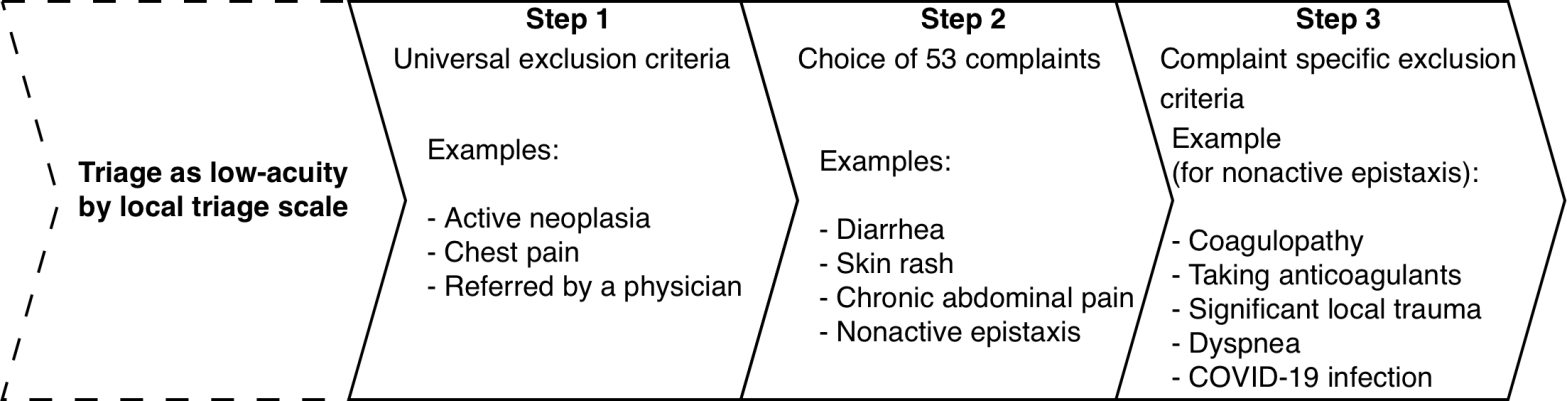
Example of the 3-step process of the electronic clinical support system for epistaxis (LGI Emergency Redirection).

The electronic clinical decision support system used in this study (LGI Emergency Redirection) was previously validated in an observational study conducted in Canada to safely redirect low-acuity ED patients [17]. The system works as a 3-step process, which can only be performed after the usual first step of the local triage protocol has been completed. Triage personnel must verify the absence of general criteria precluding redirection (eg, chest pain, active neoplasia, dialysis patients, and unstable vital signs). They then choose from a list of 53 specific minor complaints, which enables access to complaint-specific contraindications to redirection. This 3-step process is illustrated in Figure 1. If contraindications are cleared and the patient consents to redirection, an appointment within 24 hours at an alternative outpatient health care facility is made by the triage nurse.

Two main alternative care pathways are available to redirected patients. First, patients can have an appointment at 1 of 5 clinics in the regional outpatient care network directly scheduled through the LGI Emergency Redirection platform. Four of these are walk-in clinics affiliated with the same hospital group. One is a local private clinic affiliated with the regional medical care planning network. The regional on-call general practitioner is a member of the regional care network to which redirected patients can also be referred. All of the abovementioned health care providers, including GPs, have access to on-site laboratories and basic diagnostic imaging facilities. These clinics are distributed across the region of Fribourg Canton, within 2.5 to 29 km from our ED.

Second, patients may be referred to physicians outside the regional outpatient care network, including their own GP. In these cases, direct digital appointment scheduling is not supported, and appointments are coordinated through regular communication channels (eg, phone call). Once the appointment has been arranged, the exact date and time (within 24 h) are communicated to the patient before they leave the ED.

Consider the example of a 28-year-old patient presenting to the ED with a resolved epistaxis. Once admitted by dedicated staff, the patient is assessed by a triage nurse who classifies them as SETS level 4 (nonurgent). Once this triage has been completed, the nurse opens the LGI Emergency Redirection application. After ensuring that there are no universal exclusion criteria (eg, significant difficulty communicating with the patient or their family or acute mental health issues), the triage nurse selects “non-active epistaxis” in the software. The system then displays new contraindications for referral, specific to the complaint (coagulopathy, taking anticoagulants, significant local trauma, etc), to confirm eligibility. Once the patient’s eligibility has been verified and their consent for redirection obtained, the nurse uses the application’s integrated booking interface to obtain a confirmed appointment at a partner clinic within 24 hours or with a GP by telephone. The process ends with the generation of a referral document for the patient and the secure transmission of triage data to the receiving facility, thereby formalizing the transition of care.

#### Phase 1: Current Practice

**Figure 2.**
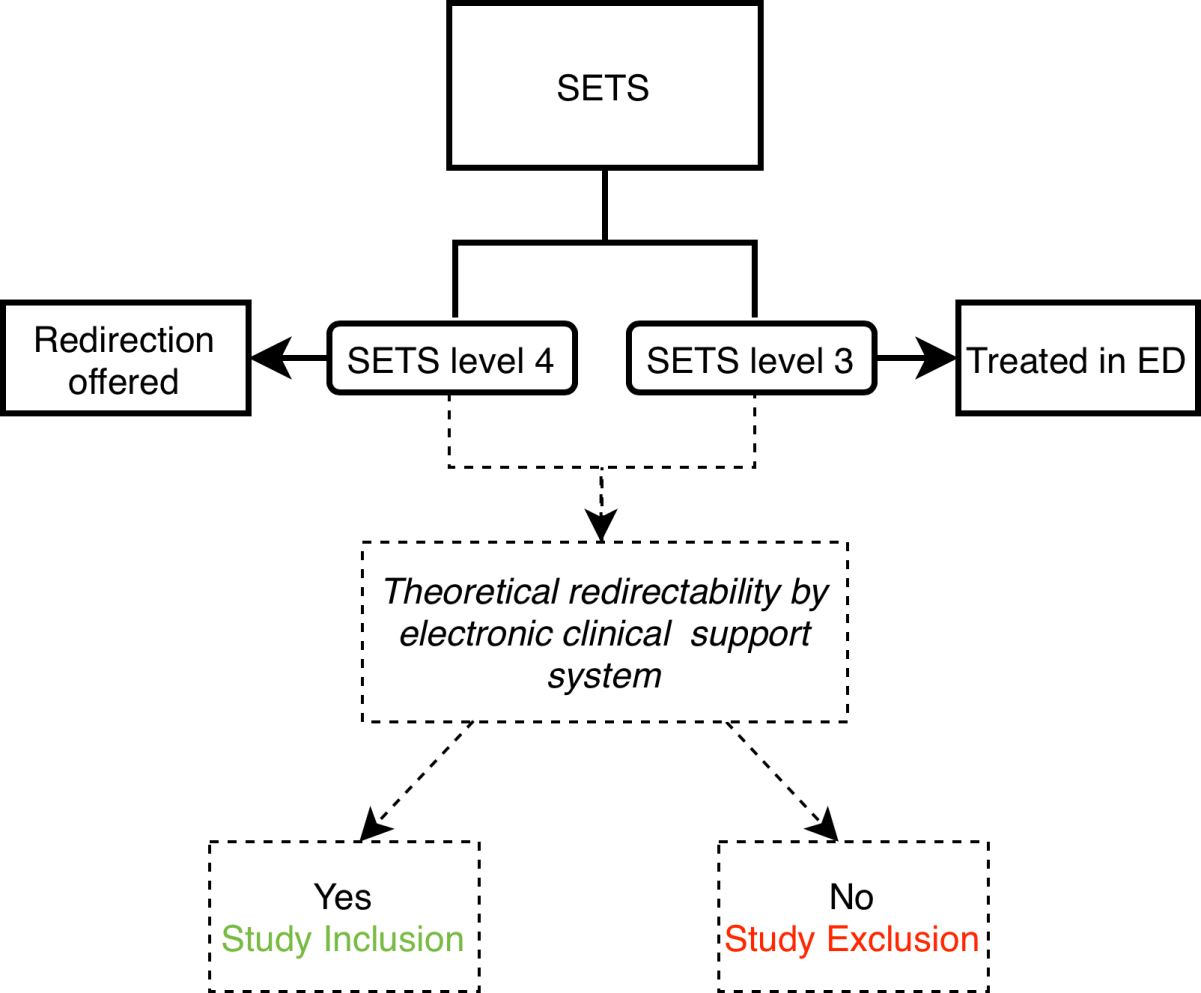
Study groups (phase 1). ED: emergency department; SETS: Swiss Emergency Triage Scale.

During the first phase, expected to last 4 to 6 months, triage and redirection of patients will follow the current practice at our ED, as described earlier. Additionally, during this phase, patients triaged as SETS levels 3 and 4 will be evaluated for their theoretical eligibility for redirection using the electronic clinical support system. At this stage, redirection will only be offered to SETS level 4 patients and will not be based on the electronic clinical support system. However, patients deemed theoretically suitable for redirection by the clinical support system will be eligible for study participation, regardless of whether redirection is performed (Figure 2). This group represents the patient population that could benefit from redirection using the electronic clinical decision support system.

This phase also allows our triage personnel to familiarize themselves with the electronic clinical support system.

#### Phase 2: Redirection of Low-Acuity Patients Using the Electronic Clinical Decision Support System

**Figure 3.**
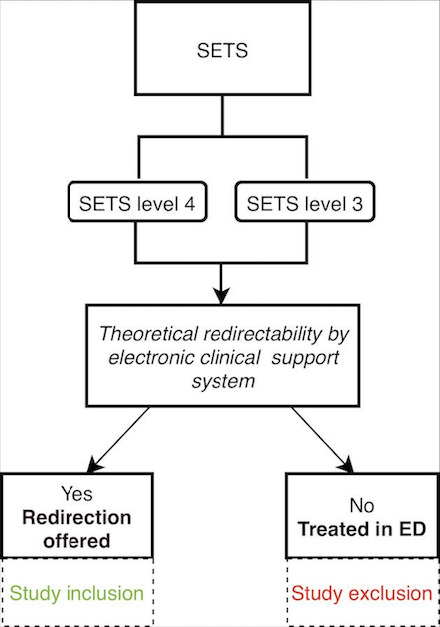
Study groups (phase 2). ED: emergency department; SETS: Swiss Emergency Triage Scale.

During the second phase, expected to last 4 to 6 months, triage and redirection of patients will be performed by combining the SETS and the electronic clinical decision support system, as illustrated in Figure 3. Patients will first be assessed for clinical acuity using the SETS. Patients classified as low-acuity (SETS levels 3 and 4) will then be assessed by the electronic clinical support system and offered redirection if they are deemed eligible. Patients will be eligible for study inclusion regardless of whether they accept redirection.

### Population and Recruitment

The inclusion and exclusion criteria are presented in Textbox 1.

Eligible patients will be identified by triage nurses after being classified as low acuity using the revised SETS and evaluated as suitable for redirection through the electronic clinical decision support system. If appropriate, the triage nurse will offer the eligible patient the option of being redirected to alternative health care providers. Patients will be informed about the study by a research assistant only after they have made their choice regarding the care pathway (acceptance of redirection or refusal and continued ED consultation).

Textbox 1. **Inclusion and exclusion criteria.**

**Inclusion criteria**

- Age ≥18 years
- Classified as low-acuity (Swiss Emergency Triage Scale [SETS] levels 3 or 4)
- Deemed eligible for redirection by the electronic clinical decision support system (LGI Emergency Redirection)
- Able to speak and read either French or German
- Oral consent obtained at day 2, followed by written informed consent

**Exclusion criteria**

- Inability to comply with study procedures (eg, cognitive impairment, significant hearing impairment without hearing aids, acute psychiatric conditions, or unavailability for telephone follow-up over the next 6 months)

### Data Collection

Baseline demographic and clinical data will be extracted from electronic medical records. Outcome data will be collected over a 6-month follow-up period, as illustrated in Table 1. Unexpected return visits to any health care facility within 48 hours of triage will be assessed via electronic medical record review and confirmed by the research team through a phone interview. Unexpected returns are defined as any medical visit related to the initial complaint and not scheduled as part of the standard discharge instructions or follow-up care plan during the initial visit. This includes unplanned consultations in any health care setting. Research assistants will conduct phone interviews with participants asking about any unexpected consultations at any health care facility (at day 2, day 7, month 1, month 3, and month 6) and their satisfaction with the redirection process (at day 2). For each study time point, our team will make 3 attempts to reach participants by phone. Health literacy will be evaluated by a written questionnaire at day 7 and month 6.

**Table 1.** Study schedule.

	Day 0 (prescreening)	Day 2 (screening)	Day 7	Month 1	Month 3	Month 6
Days	0	2	7 (+1 or −1)	30 (+4 or −4)	91 (+7 or −7)	183 (+7 or −7)
Visit	Emergency department	Phone	Phone	Phone	Phone	Phone
Eligibility	✓^a^	—^b^	—	—	—	—
Study Information	✓ (Oral and written)	—	—	—	—	—
Consent	—	✓ (Oral)	✓ (Written)	—	—	—
Confirmation of eligibility and enrollment	—	✓	—	—	—	—
Study questionnaire by phone	—	✓	✓	✓	✓	✓
HLS-EU-Q16^c^ by post or online	—	—	✓	—	—	✓

a ✓ Refers to data being collected at this timepoint.

b Refers to data not being collected at this time point.

c HLS-EU-Q16: European health literacy survey questionnaire.

### Outcomes

The primary outcome is defined as an unexpected return visit to any health care facility related to the initial complaint and occurring within 48 hours of initial triage assessment. To minimize underreporting, return visits are not restricted to our ED alone, acknowledging that patients may seek subsequent care at other EDs or with other health care providers.

Secondary study end points will include the following:

- Unexpected return visits related to the initial complaint occurring at any health care facility within 7 days of initial triage
- The number of unexpected visits related to any complaint at any health care facility reported at 1, 3, and 6 months after initial triage
- Patient satisfaction with the redirection process, as assessed by a numerical scale ranging from 0 (not at all satisfied) to 10 (perfectly satisfied)
- Patient health literacy, as assessed by the European health literacy survey (HLS-EU-Q16) questionnaire using a 4-point Likert scale, administered 7 days and 6 months after initial triage

### Handling of Missing Data

Data will be systematically screened for missing values. Exploratory analyses will assess patterns of missing data in relation to study design variables, such as time of triage, day of the week, and seasonality. Rates of participant dropout and missing data will be reported transparently. Moreover, the study duration may be extended to ensure sufficient data collection through the completion of the second phase.

### Statistical Analysis Plan

Statistical analyses will be performed using R (R Foundation for Statistical Computing) base functions if not stated otherwise. Cohen *d* values will be computed using the DescTools R package [21], and noninferiority and nonsuperiority tests will be computed using the TOSTER R package [22,23]. The significance threshold will be *P*<.05.

The season (ie, winter, spring, summer, or autumn), which affects seasonal pathologies, staffing, and workload; age; and pathology category (ie, dermatology, urology, psychiatry, etc) during triage should either be balanced between groups (electronic decision support tool vs control) or should have only a small impact on our outcomes to allow a robust interpretation of our hypotheses.

For the season and pathology categories, both groups are considered balanced if the Cramér V of the contingency table is below 0.3 (small association). For age, the groups are considered balanced if they do not differ by more than a Cohen *d* of 0.4 (small difference).

If the groups are not balanced on a given covariate, its impact on our outcomes (ie, frequency of redirection, amount of unplanned consultations, patient satisfaction, and health literacy) will be assessed using Cohen *d* values, Pearson correlations, and Cramér V values across both groups. These effect sizes should be small, that is, below 0.4 for Cohen *d* value*s* and Pearson correlations, and below 0.3 for Cramér V values.

If the groups are not balanced and the covariate has an impact on an outcome, patients who are further away from their group’s average will be excluded until the threshold for balanced groups is met.

The following hypotheses will be tested:

1. We expect patients redirected using the electronic decision support tool to have a lower or equivalent frequency of unexpected return visits in the next 48 hours and 7 days than control low-acuity patients, as indexed by a nonsuperiority test of 2 proportions with an upper bound of 5%.
2. We expect the number of unexpected visits related to any complaints in the 6-month follow-up period to be substantially lower in the electronic decision support tool group than in the control group, as indexed by a 1-sided *t* test.
3. We expect satisfaction to be noninferior in the electronic decision support tool compared with the control group, as indexed by a 1-sided *t* test with a lower margin of 2, which would correspond to the minimum amount for our 0 to 10 scale to have a shift in its interpretation.
4. We expect that the evolution of health literacy will increase significantly more in the electronic decision support tool group than in the control group, as indexed by the interaction term of a 2 × 2 ANOVA with the between-subject variable group (electronic decision tool vs control) and within-subject variable time (day 7 vs month 6). To respect the directionality of the test, the *P* value will be halved if the contrast goes in the expected direction (ie, electronic decision tool>control), and the *P* value will not be interpreted as significant if it goes into the other direction.

While the redirection network includes multiple types of facilities, interprovider variability will not be taken into account, as the study focuses on the overall safety and effectiveness of the redirection process itself rather than on the specific performance of individual partner facilities.

### Sample Size

For the first hypothesis, a priori power analysis using the pwr R package [24] indicates that 137 patients per group (274 in total) will be needed to reach 80% power for a 2-proportion test with an alpha of .05 and a smallest effect size of interest of Cohen *d*=0.3 (small). For the second and third hypotheses, 138 patients per group (276 in total) will be needed for a 1-sided *t* test with the same parameters and a smallest effect size of interest of Cohen *d*=0.3 (small). For the fourth hypothesis, a priori power analysis computed with G*Power [25] states that 99 patients per group (198 in total) will be needed for a 2 × 2 within-between ANOVA with a smallest effect size of interest of partial η^2^=0.01 (small).

On the basis of a retrospective study previously conducted in our department, we estimated that we would be able to include between 150 and 210 patients per phase, bringing the total to between 300 and 420 participants over the total study duration, which is enough to reach sufficient power for all hypotheses.

### Ethical Considerations

The clinical support system used in the study has been proven to be safe in redirecting ED patients of a university hospital, with fewer than 5% of patients returning unexpectedly [17]. The study protocol has been reviewed and approved by the Regional Ethics Committee of Vaud Canton (2023‐01655). The study will be conducted in accordance with the principles of the Declaration of Helsinki and applicable Swiss regulations. Study participation will not affect patients’ orientation or care, as they will be recruited after having accepted or declined redirection. Patients interested in participating in the study will receive a written study information and consent form at triage. On day 2, the study team will contact interested patients by phone to obtain informed oral consent. If the patient agrees, they will be included in the study and asked to return the signed informed consent form. Patients will not be compensated for participation. Each participant will be assigned a unique study identification number, and the corresponding deidentified data will be securely stored and managed using the electronic case report form system REDCap (Research Electronic Data Capture; Vanderbilt University). Study collaborators involved in data collection will have access to identifiable data, which they must maintain in strict confidence. Identifiable data will be securely stored in a locked folder within the Department of Emergency Medicine. Collaborators not involved in data collection, such as the statistical analysis team, will only have access to codified data through the electronic case report form platform. All study-related data will be archived for at least 10 years after study completion or premature termination.

## Results

This study was funded by an internal grant from Hôpital Fribourgeois in June 2024. We expect to enroll an average of 1 to 2 patients a day, 5 days a week, over a period of 8 to 12 months, for a total of 300 to 420 patients. Recruitment began in June 2025 and is expected to be completed by May 2026. As of August 2025, a total of 35 patients were enrolled. Data analysis will be conducted after completion of the 6-month follow-up period of included patients, which we expect to end in November 2026. We hope to publish the results in December 2026.

## Discussion

### Anticipated Findings

Patients redirected using the electronic decision support tool will be compared with a control group of low-acuity patients. The presence of a comparator group strengthens our study by providing data on the outcomes of the 2 cohorts and allowing us to assess the intervention’s impact.

This study is, to the best of our knowledge, the first study addressing the impact of redirection using an electronic clinical decision support system on patients’ future use of the health care system and health literacy.

However, the study presents some limitations. First, it is a single-center study conducted in an urban regional hospital that benefits from the proximity of multiple walk-in clinics offering suitable care for low-acuity conditions. This may limit the generalizability of findings to settings with fewer outpatient resources, such as rural or underserved areas.

Health literacy will be assessed using a validated questionnaire (HLS-EU-Q16) that our team has translated into the most spoken regional languages. While these translations have undergone expert review and pretesting, they have not yet been formally validated through psychometric evaluation, which may introduce measurement bias. Patient satisfaction with the redirection process will be explored using a quantitative Likert scale questionnaire. However, no qualitative component is planned, which limits our ability to capture patients’ in-depth perspectives or subjective experiences with the redirection process.

Selection bias might arise from differences in socioeconomic status or health literacy in participants accepting redirection and those refusing it. While the electronic clinical support system aims to redirect patients based on objective criteria, there might be some variability in criteria interpretation between triage nurses. In addition, outcome assessors will not be blinded to the redirection status of patients, which may introduce interviewer bias during follow-up calls. While we will follow up with patients at regular intervals, some patients might fail to report unplanned health care use, posing a risk of recall bias.

This study does not investigate the strategy’s impact on ED crowding or performance indicators, as has been assessed in the literature [16]. Furthermore, none of the study end points directly measure quality of care delivered in the ED. As such, this limits our ability to draw conclusions regarding the redirection strategy’s impact on care quality or ED overcrowding.

Management of low-acuity conditions in EDs is generally associated with higher costs compared with treatment in primary care or urgent care settings [26,27]. However, existing redirection interventions have not consistently demonstrated significant reductions in health care costs [28,29]. This study does not include cost-related end points. If the redirection strategy is found to be safe and effective, future studies should explore its economic implications, including cost-effectiveness analyses.

### Conclusions

We expect the redirection strategy guided by an electronic clinical decision support tool to be safe and result in less than 5% of unexpected returns within 48 hours of initial presentation. This approach may contribute to optimizing patient pathways by facilitating the redirection of low-acuity patients whose needs can be adequately addressed in alternative care settings, thereby allowing ED resources to remain focused on complex and acute cases. A key strength of this intervention is the standardized use of objective criteria through an electronic tool, enhancing reproducibility and consistency in clinical decision-making. Its design also facilitates transposability to other EDs if their setting permits redirection to alternative health care services.

To our knowledge, this is the first study assessing the impact of this redirection strategy on health care pathways, future unexpected consultations, and health literacy. If the redirection strategy proves to limit future unexpected visits and better health literacy, it would have a lasting impact on patient education and appropriate use of health care resources. Future research should investigate additional outcomes, including the impact of redirection on ED crowding, care quality, patient-reported experiences, and cost-effectiveness. These insights will be essential to fully understand the broader implications and scalability of the intervention.

## Abbreviations

ED: emergency department
GP: general practitioner
HLS-EU-Q16: European health literacy survey questionnaire
REDCap: Research Electronic Data Capture
SETS: Swiss Emergency Trial Scale
